# Phytotoxins Produced by *Diplodia* and Other Fungal Species Involved in Oak (*Quercus*) Diseases †

**DOI:** 10.3390/toxins18090376

**Published:** 2026-08-31

**Authors:** Antonio Evidente

**Affiliations:** Institute of Biomolecular Chemistry, Italian National Research Council (CNR), Viale Campi Flegrei 34, 80078 Pozzuoli, NA, Italy; evidente@unina.it

**Keywords:** forest plants, oak, phytopathogen fungi, phytotoxins, chemical and biological properties

## Abstract

Forests are an indispensable resource for human existence in the different regions of the world. Forests are often concentrated in a few parts and vary in size. The most important and extensive green reserve on Earth is the Amazon rainforest. Unfortunately, the forests are continually suffering massive losses due to abiotic stresses and deforestation to obtain other arable lands and microbial diseases, particularly those caused by pathogenic fungi. Pathogenic fungi play an important role in inducing oak decline, which is one of the most relevant forest diseases globally. The pathogens also affect disease development and its global distribution. In this review, the phytotoxins produced by fungal pathogens of different oak species in various world regions are described in depth. In particular, the isolation, the chemistry and biology of phytotoxins synthesized by oak pathogenic fungi are reported, and their role in pathogenesis is also discussed. For some phytotoxins, the enantioselective synthesis, the structure–biological activity relationships, and their utilization in agro-industry and medical applications are also discussed. In addition, for the *Diplodia* species, the comparison between their secondary metabolite profile and taxonomy with those of fungal pathogens of other close forest plants is discussed. Thus, fungal diseases of numerous oak trees and the serious damages they cause to the forest heritage are described, as well as the chemical and biological properties and results of some of the structure–activity relationship (SAR) studies of the phytotoxins involved. In addition, for the *Diplodia* species both phylogenetically closely related and unrelated, comparison between their secondary metabolite profile with those of pathogens of other close forest plants, in combination with traditional taxonomic characteristics, has helped to better understand the pathogenic process relationships within the same fungal f the pathogenic process within the same fungal family.

## 1. Introduction

Forests are an indispensable resource for human existence in different regions of the world. Forests are often concentrated in a few parts and vary in size. The most important and extensive green reserve on earth is the Amazon rainforest. In the Amazon, as in other tropical forests, few species spread over millions of hectares, producing fruits, seeds, or oils with high economic impact [1].

Large-scale deforestation, and particularly that of the Amazon, causes a decline in biodiversity and the availability of forest products, but these values have been largely neglected. Deforestation damages surrounding forests through increased drying of the forest floor and increased frequency of fires, reducing productivity. The loss of healthy forests causes serious losses, including carbon storage in biomass and soil, regulation of water balance and river flow, modulation of regional climate patterns, and mitigation of infectious diseases [2].

Almost all European forests have been subjected to noteworthy changes by human activity, weather events, and several biotic stressors; therefore, original forests are very rare. Some rare patches of original forests can be found in mountainous regions of Central and Eastern Europe, the Far North of Europe, and the boreal European part of Russia. Over the centuries, the different uses of forests have modified land use and landscapes and have generated their fragmentation, the very structure of the forest zones, and historical and monumental sites [3].

Extreme climate events will increase the frequency and intensity of stress on forests, significantly impacting their health. Various fungal tree diseases could become more devastating due to various factors. Drought and flooding make trees more susceptible to pathogen attack, rising temperatures and humidity influence the sporulation and dispersal of pathogens, and changes in climate could favor some of them. Furthermore, pathogen migration triggered by climate change may increase the incidence or geographic spread of diseases when pathogens encounter new hosts and/or potential vectors, and new threats may emerge due to changes in tree species composition or invasive species [4]. Thus, in addition to abiotic stresses and deforestation to obtain other arable lands, microbial diseases, particularly those caused by pathogenic fungi, cause significant damage to the forestry heritage, which spreads undergrowth species, especially in reforestation practices. Severe economic losses are also suffered by timber-producing countries and associated industries, including paper mills.

Oak decline is a significant problem in oak forests all over the world. Many factors contribute to this phenomenon, but the one due to fungal diseases is significant. Fungal diseases have caused the death of millions of oak trees around the world [5,6], having a significant negative impact on wildlife, for which the oaks are a habitat and food source [7]. Fungal diseases of oaks have been observed since the mid-1700s with high frequency throughout the world from the 1980s onwards [8], because they are considered one of the main challenges to environmental management in large areas such as the Mediterranean basin [9]. Fungi that cause oak decline include atrophied leaves and extensive necrosis, dead branches, death of root tips, dead suckers, localized necrosis in the bark and phloem, local death and discoloration of the sapwood, and dead trees. In total, 80 species of fungi, mainly belonging to the Ascomycetes and Deuteromycetes, have been found among oak pathogens. Different fungal species have been more frequently associated with the same type of symptoms than others [10]. A recent review [11] reports *Phytophthora* species and latent pathogens, including some species of *Botryosphaeriaceae*, among the most significant biotic stressors for European oaks. Among them, *Biscogniauxia mediterranea*, the causative agent of black canker, and the oak anthracnose fungus *Apiognomonia quercina* have been highlighted. Other biotic stressors include powdery mildew fungi of the genus *Erysipe*, wood-rotting fungi such as *Armillaria* spp. and other basidiomycetes [11].

This review reports briefly the classification of *Quercus* plants and extensively the isolation, chemistry and biology of phytoxins synthesized by their pathogenic fungi, discussing their role in pathogenesis. Microbial competition, the structure–biological activity relationship, and the potential applications of some of these phytotoxins in agriculture and medicine are also discussed. Furthermore, for the *Diplodia* species, the comparison between their secondary metabolite profile and taxonomy with those of fungal pathogens of other close forest plants is reported.

## 2. Fungal Phytotoxins

### 2.1. Diplodia spp.

Fungi belonging to the genus *Diplodia* are widespread worldwide and are known as pathogenic, endophytic, and latent pathogens of a variety of woody hosts [12,13,14,15]. The pathogenic species induce various symptoms on host plants, including shoot wilting, desiccation, cankers, and fruit rot. Some *Diplodia* species have a wide host range, such as *Diplodia seriata*, which has been reported on over 250 hosts [16]. On the other hand, some species appear to be restricted to a single host or to closely related genera. For example, *Diplodia pinea* and *Diplodia scrobiculata* have been isolated only from conifers, while *Diplodia corticola* and *Diplodia cupressi* have been reported only on *Quercus* and *Cupressus* species, respectively [17].

Several investigations have been performed on fungi of the *Diplodia* and *Neofusicoccum* genera, as well as studies on their role in the pathogenic process of oak decline in the Mediterranean basin [18,19,20,21]. The increased wilting of oak in Europe, North Africa and North America has stimulated research worldwide to elucidate the physiological processes involved in this disease. *Diplodia corticola*, in respect to the other *Diplodia* spp. infecting oaks, is the fungus that has caused the most severe damage to both the vitality and productivity of trees and consequently a negative impact on oak ecosystems [22,23,24,25].

**Figure 1:**
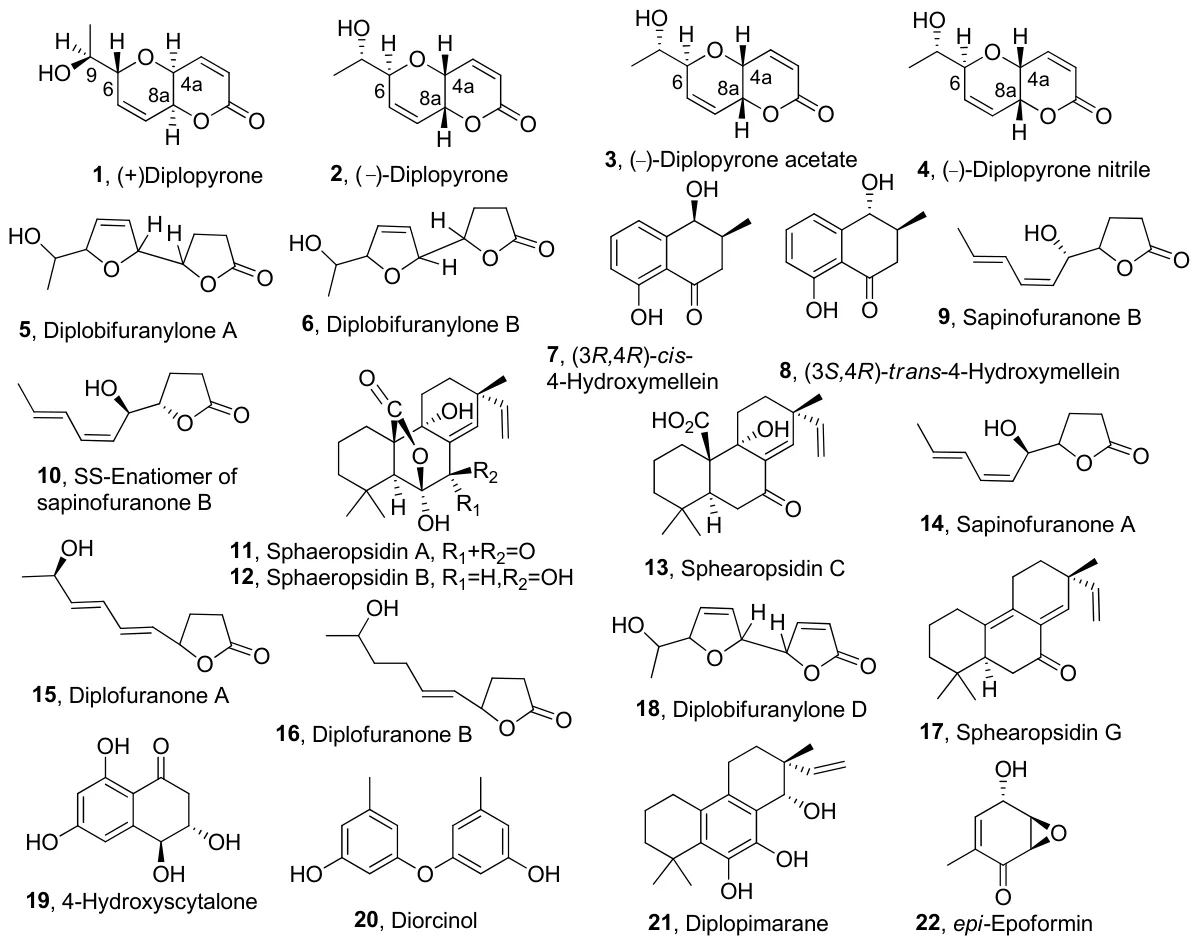
Phytotoxins produced by *Diplodia mutila*, *Diplodia corticola* and *Diplodia quercivora*.

*Diplodia mutila* [26], widespread in the oak forests of Sardinia Island (Italy), is an endophyte responsible for the cork oak (*Quercus suber* L.) decline [27]. The fungus caused symptoms very similar to the tracheomycotic ones and can infect trees of different ages. When inoculated in the stems of young cork oak plants, the fungus caused a slight collapse, and around the inoculation site, a dark brown discoloration of the cortical tissues was observed. An immediate wilting of the overlying plant and consequently the development of secondary shoots below it were observed [28]. These symptoms suggested the ability of the fungus to produce phytotoxic metabolites, as also observed for *D. mutila* isolates from cypress or other oak species [29]. Diplopyrone (**1**, Figure 1, Table 1), the main phytotoxin, was produced by *D. mutila* in liquid cultures and identified as a novel monosubstituted tetrahydropyranpyran-2-one. Its absolute configuration of carbon (C-9) was also assigned as *S* [26]. Pyran-2-ones are widely distributed in nature, being metabolites of plants, animals, marine organisms and microbes. Many of the latter possess interesting biological properties [30,31,32]. The pyran-2-one moiety characterizes other secondary metabolites synthesized by fungi belonging to different genera, including *Alternaria*, *Aspergillus*, *Fusarium* and *Trichoderma*. These metabolites have shown antibiotic, antifungal, cytotoxic, neurotoxic and phytotoxic activities [33,34]. Diplopyrone showed significant phytotoxicity on oak at concentrations ranging from 0.01 to 0.1 mg/mL, while necrotic lesions on leaves within 4 days of administration were observed at concentrations ranging from 0.001 to 0.1 mg/mL. Compound **1** caused cork cuttings to wilt within 8 days. Compound **1** assayed on tomato cuttings (a non-host plant), at concentrations of 0.1 and 0.2 mg/mL, induced phytotoxic effects with the collapse of internal stem tissues. No phytotoxicity was detected at lower concentrations [26,33].

Subsequently, the absolute stereochemistry 6-[(1*S*)-1-hydroxyethyl]-2,4a(*S*),6(*R*), 8a(*S*)-tetrahydropyrano [3,2-b]pyran-2-one was determined for (+)-diplopyrone (**1**). This non-empirical assignment was obtained using two different methods: (a) circular dichroism (CD) spectroscopy and (b) *ab initio* calculation of the optical rotatory power [29].

The synthesis of the enantiomer of (+)-diplopyrone (**2**, Figure 1) was realized starting from a derivative of D-galactose, which is commercially available. The highly stereoselective pyranose chain extension was obtained by preparation of a vinyl glycoside via Isobe C-alkynylation-rearrangement/reduction, and RCM (Ring-closing metathesis)-based pyranopyran constructions were the main steps of this synthesis. Two derivatives of the enantiomer **2** were prepared by acetylation and conversion of its secondary hydroxy group into a nitrile (**3** and **4**, respectively, Figure 1, Table 1). Pyranpyrones **2**, **3**, and **4** showed bacteriocidal activity, and the derivative **4** showed toxicity towards *Edwardsiella ictaluri* and *Flavobacterium columnare*, common fish bacterial pathogens, which induce, in catfish, enteric septicemia (ESC) and columnaris disease, respectively. Compared to a commercial antibiotic, compound **4**, tested in vitro, was significantly more toxic against *E. ictaluri*. The same derivative also showed phytotoxic activity on *Lemna paucicostata* (duckweed), confirming the potential of these pyranopyrones as antibiotics and herbicides [39].

The endophytic fungus *Diplodia corticola* (anamorph form of *Botryosphaeria corticola*) is widespread in Sardinian oak forests and represents another pathogen of cork oak [27]. In fact, the fungus, infecting plants of different ages, caused symptoms including dieback, cankers, and vascular necrosis. Diplobifuranylones A and B (**5** and **6**, Figure 1, Table 1), two 5′-monosubstituted tetrahydro-2*H*-bifuranyl-5-ones, were purified from the organic extract of fungal culture filtrates [29]. Compounds **5** and **6** were obtained together with (+)-diplopyrone (**1**), (3*S*,4*R*)-t*rans*- and (3*R*,4*R*)-*cis*-4-hydroxymellein [29,40,41,42], sapinofuranone B and its (*S*,*S*)-enantiomer (**7**–**10**, Figure 1, Table 1); the latter (**10**) was previously isolated from *Acremonium strictum*.

*D. corticola* is a saprophytic fungus found on plant surfaces and soil [35]. In addition, sphaeropsidins A–C (**11**–**13**, Figure 1, Table 1) were also produced from this fungus [29]. Compound **9**, characterized as 4-[(2*Z*,4*E*)-1-hydroxy-2,4-hexadienyl]butan-4-olide, was isolated together with its C-1 epimer, named sapinofuranone A (**14**, Figure 1, Table 1), from *Sphaeropsis sapinea*, a pathogen collected from *Cupressus macrocarpa*, which caused a wide range of disease symptoms in conifers [29]. Diplobifuranylones A and B (**5** and **6**) are two diastereomeric 5′-(1-hydroxyethyl)-3,4,2′,5′-tetrahydro 2*H*-[2,2′]bifuranyl-5-ones, and their relative configuration was assigned by NOESY and ROESY NMR spectra. The absolute configuration of the chiral carbon of the hydroxyethyl side chain at C-5′ was assigned by applying Mosher’s method [43]. It proved to be *S* and *R* in **5** and **6**, respectively. Diplobifuranylones A and B were not toxic to tomato non-host plants with respect to the strong significant phytotoxicity of related sapinofuranones A (**14**) and B (**14** and **9**) on host (pine and cypress) and non-host plants. The lack of phytotoxicity of **5** and **6** could be due to the structural modification of the 1-hydroxy-2,4-hexadienyl side chain at C-4, which in **5** and **6** appeared to be, respectively, a *trans*- and *cis*-2,5-disubstituted 2,5-dihydrofuran ring, while the γ-lactone moiety did not change [29]. Diplobifuranilones belong to the bisfuranoids and showed other similarly significant biological activities as the aflatoxins [44]. They are broadly present in nature as substances synthesized by *Aspergillus*, *Bipolaris*, *Cercospora*, *Chaetomium*, *Dothistroma*, *Farrowia*, and *Monocillium*. Diplobifuranilones have constituted by two furanoid rings linked through one side, while in bisfuranyls such as **5** and **6**, the two rings are joined by a bond, as in the first example reported among natural compounds. These kinds of bisfuranyls were previously only known as synthetic compounds [45,46,47]. Successively, from *D. corticola*, diplofuranones A and B (**15** and **16**, Figure 1, Table 1), two 4-monosubstituted 2(3*H*)-dihydrofuranones, were isolated too. The diplofuranone A (**15**) was characterized as 4-[(1*E*,3*E*)-5-hydroxyhexadienyl]butan-4-olide, while diplofuranone B (**16**) was characterized as its corresponding 3,4-dihydro side chain derivative [29]. The configuration of the hydroxylated secondary carbon, contained in the side chain of diplofuranone A, was assigned as *R* by applying Mosher’s method [43]. Diplofuranone A (**15**) did not exhibit phytotoxicity when assayed on a non-host tomato plant at 0.2 mg/L. Diplofuranone B was not assayed as the available amount was not sufficient for the test. The lack of toxicity of **15**, compared to the phytotoxic activity of sapinofuranones A and B (**14** and **9**), did not come as a surprise, as compound **16** has strongly modified the side chain at C-4, suggesting the importance of this structural feature when imparting the phytotoxicity [29]. Diplofuranones, as previously cited above, are phytotoxins produced by *S. sapinea* infecting cypress, while the *S,S*-enantiomer of sapinofuranone B was isolated from *A. strictum*. Sapinofuranones A and B (**9** and **14**) were characterized as 5-((*R*,2*Z*,4*E*)-1-hydroxyhexa-2,4-dien-1-yl)dihydrofuran-2(3*H*)-one and are epimers of compound **14** at the hydroxylated carbon of the side chain, respectively [29]. Butanolides are scarcely present in nature and are closely related to butanolides. The latter were already known as metabolites of plants, fungi and lichens and also showed interesting biological activity [44]. Among them there are seiridines, which are phytotoxic 3,4-dialkylbutenolides produced by three *Seiridium* species associated with cypress canker [29].

Finally, from organic extracts of *D. corticola* culture filtrates, sphaeropsidin G and diplobifuranylone D (**17** and **18**, Figure 1, Table 1), a new *nor*-pimarane diterpene and a new monosubstituted bifuranylone, respectively, were isolated together with 4-hydroxyscytalone and diorcinol (**19** and **20**, Figure 1, Table 1). Sphaeropsidin G and diplobifuranylone D were identified as 1,1,7-trimethyl-7-vinyl-2,3,4,4a,4b,5,6,7,10,10a-decahydro-1*H*-phenanthren-9-one and 5′-(1-hydroxyethyl)-2′,5′-dihydro-2*H*-[2,2′]bifuranyl-5-one, respectively. The relative configurations of sphaeropsidin G and diplobifuranylone D were assigned by comparison of their spectroscopic properties with those of sphaeropsidin A and diplobifuranylones A–C, respectively, and confirmed by NOESY spectra [29]. Each compound was tested for phytotoxicity, antifungal properties, antioomycetes, and zootoxicity. At a concentration of 1 mg/mL and using the leaf puncture bioassay evaluated on holm oak (*Quercus ilex*), *Quercus afares*, Algerian oak (*Quercus canariensis*) and European hackberry (*Celtis australis*) leaves, none of the compounds, except compound **20**, were phytotoxic. In particular, diorcinol (**20**) caused necrotic lesions, although with different lesion area sizes on all plant species tested. The data on the antifungal activity of the isolated compounds, tested on three plant pathogenic fungi (*Athelia rolfsii*, *Fusarium avenaceum* and *Lasiodiplodia mediterranea*), were very similar to those observed for their phytotoxicity, whereas the antioomycete activity, tested on *Phytophthora cinnamomi* at concentrations of 0.05, 0.01 and 0.2 mg/plug and using PCNB, Toclofos methyl and Metalaxyl-M as positive controls, appeared to be species-dependent. In fact, only compound **20** showed significant activity towards all plant pathogens tested, among which *A. rolfsii*, *L. mediterranea* and *P. cinnamomi* appeared to be more sensitive (100% inhibition rate at all concentrations) to this compound, and *F. avenaceum* was found to be less sensitive. All the compounds were tested at concentrations from 5 to 250 µg/mL in the brine shrimp (*Artemia salina* L.) larvae bioassay, which is widely used for toxicology and ecotoxicology studies. Compound **18** was not toxic to *A. salina* at the highest concentration tested. Compounds **17** and **20** had significant toxicity (LC_50_ (lethal concentration, 50%) values of 11.0 and 20.1 µg/mL, respectively), while compound **19** was found to be less toxic with LC_50_ 90.6 µ/mL [29].

*Diplodia quercivora*, a more recently described oak (*Quercus canariensis*) pathogen originally found on declining trees in Tunisia, was also shown to produce phytotoxins in vitro. Among these, the main one was diplopimarane (**21**, Figure 1, Table 1), a new 20-*nor-ent*-pimarane, but there were also sphaeropisdins A (**11**) and C (**13**), and (+)-*epi*-epoformin (**22**, Figure 1, Table 1), a known cyclohexene oxide. Diplopimarane was characterized as (1*S*,2*R*)-2,8,8-trimethyl-2-vinyl-1,2,3,4,5,6,7,8-octahydrophenanthrene-1,9,10-triol and used to prepare some of its derivatives such as 6-*O*-methyl, 6,7-*O*,*O*′-dimethyl, 6,7,14-*O*,*O*′,*O*″-triacetyl, 7-*O*-*p*-Br-benzoyl and 15,16-dihydro derivatives [29]. The relative and absolute *S* and *R* configurations at C-14 and C-13 of diplopimarane were determined, respectively, by X-ray analysis and by applying Mohser’s method [43] to its 6-*O*-methyl derivative. Diplopimarane showed different activities, including remarkable phytotoxicity on host (*Quercus ilex* L.), cork oak (*Q. suber* L.), and tomato non-host plant (*Lycopersicum esculentum* Mill.) leaves. Among its derivatives, the 6-*O*-methyl and 15,16-dihydro ones, tested on tomato cuttings, showed, to different extents, stunting on the stem at 0.2 and 0.1mg/mL. Diplopimarane was also tested in an in vitro assay for antifungal and antioomycete activity against four plant pathogens. Among these pathogens, *Phytophthora cinnamomi* and *Phythophtora plurivora* seemed to be the most sensitive target organisms, followed by *Athelia rolfsii*, whereas *D. corticola* appeared to be the most resistant one [29]. In testing the zootoxicity using *Artemia salina* bioassay, diplopimarane (**21**) and its derivatives caused more than 50% larval mortality at 200 μg/mL after 36 h of exposure to the metabolites [29].

(+)-*Epi*-Epoformin (**22**) was also synthesized by an unidentified fungus collected from a diseased leaf of *Lagerstroemia indica* (*Lagerstroemia indica* L. Pers.) [48]. The enantioselective and asymmetric total synthesis of toxin **22** was also reported [49], and the assignment of its absolute stereochemistry was determined by time-dependent density along with comparison of that of other naturally occurring cyclohexene oxides [29]. Compound **22** possesses several interesting biological activities, including antifungal, zootoxic, and phytotoxic activity. In particular, it caused inhibition of lettuce seed germination. The structural characteristics of **22** prompted a structure–activity relationship study, preparing eight key hemisynthetic derivatives, which showed the acetylation or the oxidation of the hydroxy group, the reduction of the carbonyl group, the opening of the epoxide in the corresponding diastereomeric triols or the corresponding iodohidryn, the saturation of the double bond, and the reductive opening of the epoxy group to yield the corresponding diastereomeric diols. These derivatives were tested in comparison with **22** and the commercial herbicide Logran in the bioassay of etiolated wheat coleoptile. Almost all compounds inhibited the growth of wheat coleoptile, showing comparable to or greater activity than that of (+)-*epi*-epoformin and the reference herbicide Logran. The carbonyl group proved to be essential for phytotoxic activity, as well as appeared to be the conjugated double bond, while modifications of the epoxide group had no effect on phytotoxicity [50].

Studies conducted on the secondary metabolic profiles of *Diplodia* species, and those of oak pathogenic species described in detail above, are very useful when combined with traditional fungal taxonomy to better understand the differences between species belonging to the same fungal genus. In addition to oak pathogenic *Diplodia* species, we can also add knowledge of the metabolites produced by *Diplodia* species pathogenic to other important forest trees (pine, juniper, and ash).

#### 2.1.1. *Diplodia africana*

**Figure 2:**
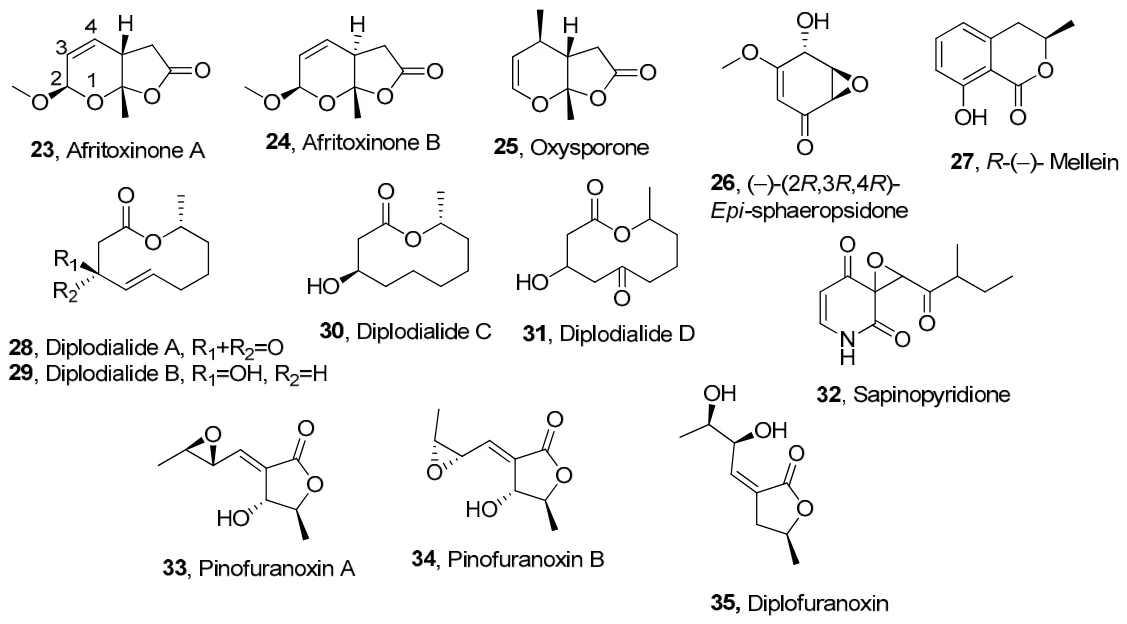
Phytotoxins produced by *Diplodia africana*, *Diplodia sapinea* and *Diplodia subglobulosa*.

Afritoxinones A and B (**23** and **24**, Figure 2, Table 1), two phytotoxic dihydrofuropyran-2-ones, were isolated from *Diplodia africana*, the causal agent of branch dieback of Phoenician juniper (*Juniperus phoenicea* L.) in Italy. Such a plant is a typical juniper diffused in the Mediterranean basin. In Sardinia (Italy), it grows mostly at low altitudes close to the coast and in particular on the small islands of the La Maddalena Archipelago, including Caprera Island [51]. Afritoxinones A and B (**23** and **24**) were characterized as (3a*S*,6*R*,7a*S*)-6-methoxy-7a-methyl-3,3a,6,7a-tetrahydro-2*H*-furo [2,3-b] pyran-2-one and its 3a-epimer, respectively. The same fungus also produced spheropsidin A (**11**) and oxysporone, (–)-(2*R*,3*R*,4*R*)-*epi*-spheropsidone, *R*-(–)-mellein (**25**, **26** and **27**, Figure 2, Table 1), and (3*R*,4*R*)- and (3*R*,4*S*)-4-hydroxymelleins (**7** and **8**) [29]. The phytotoxicity of metabolites **23**–**25** was evaluated using different bioassays. Oxysporone (**25**) at concentrations between 0.1 and 1 mg/mL showed significant toxicity on holm oak, cork oak, and tomato leaves, inducing necrosis (sized between 2 and 30 mm^2^) on the tested species. On the same species, afritoxinones A and B showed a marked decrease in activity at concentrations of 1.0 and 0.5 mg/mL, and at lower concentrations, they were not phytotoxic. Oxyporone also induced phytotoxicity on tomato cuttings when assayed at 0.2 and 0.1 mg/mL, causing complete wilting 48 h after application. Compound **25**, tested at 0.05 mg/mL, induced the lowest stem burn. Compounds **23** and **24** induced no symptoms. Similarly, compound **25** tested at the same concentrations caused yellowing and browning of leaves and drying of cut twigs of *Phoenicean juniper*. These symptoms were very similar to those observed in the field on the same plant infected by *D. africana*, showing compound **25** as a virulent or pathogenicity factor, while compounds **23** and **24** were consistently found to be less phytotoxic. The unsubstituted γ-lactone moiety is present in all three compounds (**23**–**25**), so the decrease in phytotoxicity of compounds **23** and **24** is likely due to the modification of the functionality of their dihydropyran ring. Indeed, both afritoxinones **23** and **24**, compared to oxysporone, have at C-2 a methoxy instead of the hydroxyl group, with a consequent shift in the double bond from C(2)–C(3) to C(3)–C(4), and dehydroxylation of the C-4. Furthermore, upon inspection of Dreiding models, these modifications do not result in a different stereochemistry of the dihydropyran ring. The effect on the phytotoxic activity of the modification of the junction between the two rings, as well as that of the γ-lactone ring, should be further studied by preparing suitable derivatives of oxysporone [29].

#### 2.1.2. *Diplodia sapinea*

*Diplodia sapinea* is one of the most widespread conifer pathogens worldwide, with significant economic impacts, particularly on the timber-producing industries. Typical symptoms of the fungal disease include blossom-end rot, resinous cankers on the main stem and branches, desiccation, and a bluish stain in the sapwood. Abiotic stresses favor *D. sapinea* infections, which have been found to be more severe in the Southern hemisphere than in the Northern one [52]. The fungus has been reported in the Baltic countries [38], but also in the Mediterranean basin, particularly in Tunisia and Algeria [53]. *D. sapinea* is a known producer of phytotoxins that play a significant role in host plants [54]. However, all knowledge on virulence factors involved in the pathogenesis process and their biochemical targets is still limited [29]. Among the phytotoxins produced by *D. sapinea* are diplodialides A–D (**28**–**31**, Figure 2, Table 1), isolated for the first time in 1975 during the studies on steroid hydroxylase inhibitors. Compound **28** at 125 p.p.m showed the inhibition of 11α-hydroxylase in *Rhizopus stolonifera*, which is commonly known as black bread mold [55,56]. Later, the above cited sapinofuranones A and B (**9** and **14**), sapinopyridione (**32**, Figure 2, Table 1), were isolated from three strains of *D. sapinea* (syn *Sphaeropsis sapinea*) collected from symptomatic cypress (*Cupressus sempervirens* L. and *Cupressus macrocarpa* Hartw) in Italy [29], whereas *R* (−)-mellein (**27**), as well as (3*R*,4*R*)- and (3*R*,4*S*)-4-hydroxymellein (**7** and **8**), were obtained from a Sardinian strain of *S. sapinea* obtained from *Pinus radiata* [29]. Sapinopyridolone (**32**), a 2,4-trisubstituted pyridione, was obtained in high yield only from the *S. sapinea* strain collected from *Cupressus sempervirens. S. sapinea* is a worldwide-diffused fungal pathogen of conifers. Sapinopyridione was identified as 6-methyl-2-(2-methyl-1-oxobutyl)-1-oxa-5-azaspiro [2.5]oct-6-ene-4,8-dione, and its structure was confirmed through the preparation of three key derivatives, such as the 9,*O*-dihydro derivative and its two 3-substituted 4-hydroxy-6-methyl-2-pyridones. The phytotoxic and antifungal activities of these derivatives were tested on host plants and on three *Seiridium* species, respectively, and compared to those of compound **32**. From the point of view of a SAR study, both for phytotoxicity and antifungal activity, the pyridine and oxirane rings appeared to be important structural factors. The carbonyl group of the side chain also appeared to play a role in conferring activity [29].

#### 2.1.3. *Diplodia pinea*

From the organic extract of *D. pinea*, isolated from a cankered branch of maritime pine (*Pinus pinaster*) collected in a declining stand located in northwest Tunisia, pinofuranoxin A and B (**33** and **34**, Figure 2, Table 1), two bioactive trisubstituted furanones, were purified [29]. They were identified as (4*R*,5*S*,*E*)-4-hydroxy-5-methyl-3-(((2*R*,3*R*)-3-methyloxiran-2-yl)methylene)dihydrofuran-2(3*H*)-one and its diastereomer with inverted configuration of the epoxy group. Pinofuranoxins A and B tested on ivy, bean, and holm oak leaves at a concentration of 1 mg/mL caused necrotic lesions on all tested plants, resulting in lesion areas of 112, 46, and 61, 79, 55, and 50 mm^2^, respectively. Compound **33** also induced necrosis at 0.5 and 0.1 mg/mL, while compound **34** did not exhibit phytotoxic activity at 0.1 mg/mL on all plant species. Both pinofuranoxins (**33** and **34**) were also tested against two phytopathogenic fungi (*Athelia rolfsii* and *Diplodia corticola*) and the oomycete *Phytophthora cambivora*, using pentachloronitrobenzene (PCNB) and metalaxyl-M as positive controls. Compound **33** completely inhibited mycelial growth at concentrations of 0.2 and 0.1 mg/cap. When tested against brine shrimp (*Artemia salina* L.) at 200 μg/mL, pinofuranoxins A and B caused 96% and 51% larval mortality, respectively, while at the other two concentrations (100 and 50 μg/mL), larval mortality was less than 20% for compound **33**, while compound **34** was inactive [29]. While dihydrofuranones and epoxides are well known as natural compounds [44] and also as fungal phytotoxins [57], pinofuronoxins A and B (**33** and **34**) exhibit a truly unique combination of functional groups. They have shown similar phytotoxic activity, but different antifungal and zootoxic activity. Both the α,β-unsaturated carbonyl (probably involved in nucleophilic Michael addition) and the epoxy ring (probably involved in nucleophilic substitution), present in the two toxins, are well-known structural features often associated with biological activities [57]. The different antifungal and zootoxic activity of the two pinofuranixins could be attributed to the change in the absolute configuration of the epoxy ring [57].

#### 2.1.4. *Diplodia subglobosa*

*Diplodia subglobosa* has been a species associated with a narrow host range and has a restricted geographical distribution in Europe, differentiating itself from several fungal genera infecting ash (*Fraxinus excelsior* L.) [37,58]. *D. subglobulosa* has been an emerging pathogen involved in the etiology of ash dieback, a disease that has been the major threat to the European ash heritage and the associated timber industry. The fungus has been phylogenetically related to *D mutila*, *D. africana* and *Diplodia olivarum*, which are three polyphagous species able to synthesize a plethora of bioactive compounds in vitro, including several phytotoxins [29]. *D. subglobulosa* produced in vitro diplofuranoxin (**35**, Figure 2, Table 1), a novel disubstituted dihydrofuranone, along with spheropsidins A and C, *R*-(−)-mellein, (3*R*,4*R*)- and (3*S*,4*R*)-4-hydroxymelleins, and (–)-(2*R*,3*R*,4*R*)-*epi*-spheropsidone (**11**, **13**, **27**, **7**, **8**, and **26**) [29]. Diplofuranoxin (**35**) has three stereocenters and thus can exist as eight possible stereoisomers. The relative and absolute configurations (3Z,5S,7S,8S) for metabolite **35** were determined by combining NOESY NMR experiments and computational analysis of the electronic circular dichroism spectrum (ECD), and thus compound **35** was characterized as (*S*,*Z*)-3-((2S,3*R*)-2,3-dihydroxybutylidene)-5-methyl dihydrofuran-2(3*H*)-one. The phytotoxic activity of all metabolites was tested by leaf-puncture bioassay at a concentration of 0.4 mg/mL. When assayed on *Phaseolus vulgaris* leaves, sphaeropsidin A and (–)-(2*R*,3*R*,4*R*)-*epi*-sphaeropsidone (**11** and **26**) were toxic at different concentrations, while none of the other compounds showed phytotoxicity. Furthermore, sphaeropsidin A (**11**) was assayed at concentrations of 0.2 and 0.02 mg/plug and used Metalaxyl-M as a positive control against *Phytophthora cambivora*, which caused the “ink disease” on chestnut, completely inhibiting fungal mycelial growth. When testing (–-(2*R*,3*R*,4*R*)-*epi*-spheropsidine (**26**), only partial inhibition (37% at a concentration of 0.2 mg/plug) was observed. The other metabolites were not toxic. When these metabolites were tested towards brine shrimp (*Artemia salina* L.) larvae at 50 μg/mL, no toxicity was similarly observed [29].

Based on the results shown above, it can be concluded that spheropsidin A is produced by many *Diplodia* species, including *D. quercivora*, *D. corticola*, *D. mutila*, *D. cupressi*, and *D. globulosa*, while it was produced by species such as *D. africana* and *D. sapinea*. Melleins were obtained from a large number of *Diplodia* species. Some other metabolites were produced by a single species, such as diplobifuranylones isolated from *D. corticola*, diplopimarane from *D. quercivora*, spheropsidones from *D. cupressi*, oxysporone and afritoxinones A and B from *D. africana*, and diplodialides and sapinopyridione from *D. sapinea*. Diplopyrone was isolated only from *D. mutila* and *D. corticola*. The results obtained expand our knowledge of the secondary metabolic profile of the studied *Diplodia* species and show that some metabolites appear to be produced only by certain *Diplodia* species, both phylogenetically closely related and unrelated.

### 2.2. Biscognauxia spp.

#### 2.2.1. *Biscognauxia mediterannea*

**Figure 3:**
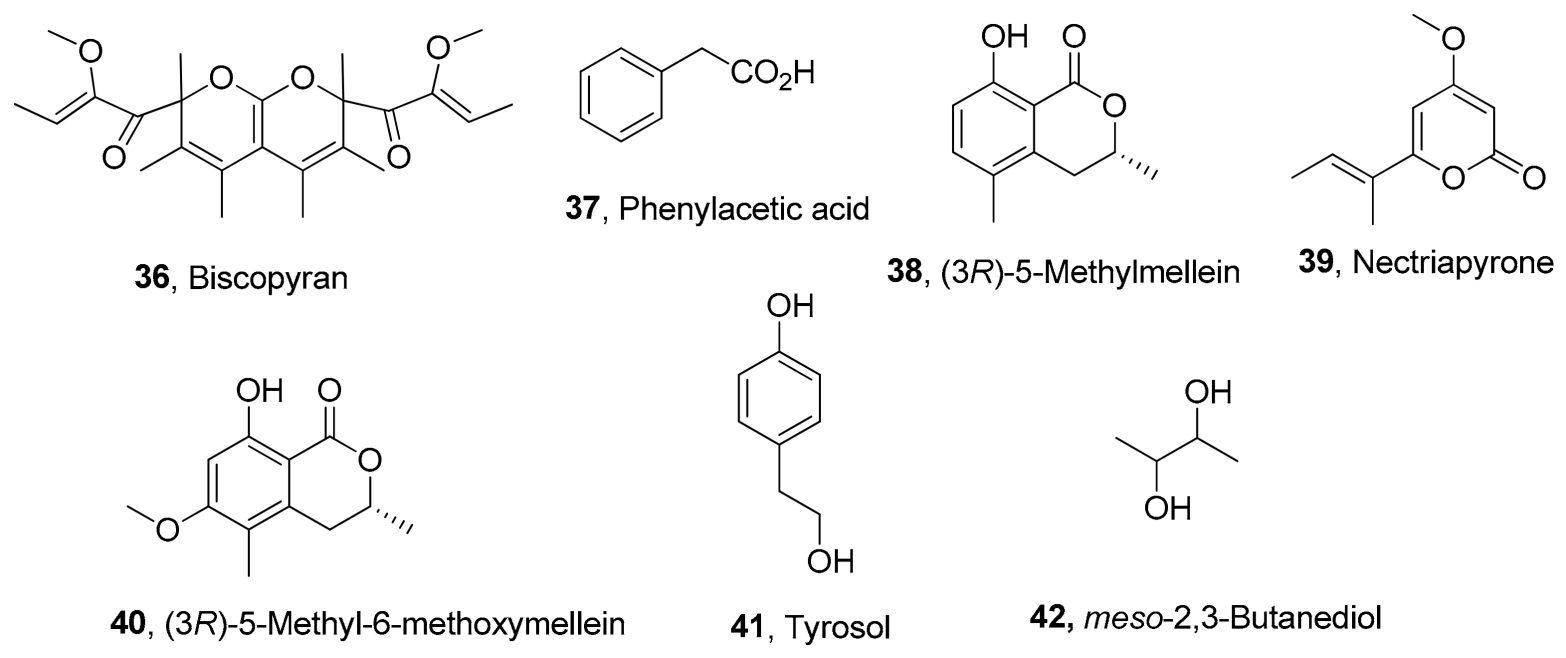
Phytotoxins produced by *Biscognauxia mediterranea* and *Biscognauxia rosacearum*.

Biscopyran (**36**, Figure 3, Table 2), a new phytotoxic hexasubstituted pyranopyran, was isolated from *Biscogniauxia mediterranea* along with phenylacetic acid 5-methylmellein (**37** and **38**, Figure 3, Table 2). The three metabolites were characterized as (*Z*)-2-methoxy-1-[7-((*Z*)-2-methoxybut-2-enoyl)-3,4,5,6-tetramethyl-2*H*,7*H*-pyrano [2,3-b]pyran-2-yl]but-2-en-1-one, 2-phenylacetic acid, and (*R*)-8-hydroxy-3,5-dimethylisochroman-1-one, respectively [29]. *B. mediterranea* is a fungal endophyte diffused in Sardinia oak forests, and has been recognized as one of the agents that causes the decline of oak (*Quercus suber* L.) [28]. The endophyte can survive in all aerial organs of host plants and then acts as an opportunistic pathogen when oaks undergo prolonged periods of stress. *B. mediterranea* induces discoloration of woody tissue, desiccation, and cankers of the trunk and branches, progressing to the appearance of characteristic black-carbonaceous stromata on dead organs [59]. Both biscopyran and phenylacetic acid showed non-selective phytotoxic activity. Compound **37** showed phytotoxicity on oak at concentrations between 0.26 and 0.026 mM 5 days after absorption of the tested solution, while necrosis appeared on leaf surfaces and cuttings of the same plant wilted within 10 days after absorbing a solution at 0.26 mM.

The same compound **37**, tested on cut tomato seedlings, caused phytotoxicity at concentrations between 0.26 and 0.13 mM, resulting in collapse of the internal tissues of the stem. Biscopyran (**36**) induced epinasty on cork oak cuttings when tested at concentrations between 0.26 and 0.026 mM, while on tomato, it caused wilting at concentrations between 0.13 and 0.26 mM.

In Portuguese cork oaks, severe infestations of the ambrosia beetle *Platypus cylindrus* have been reported for three decades, firstly on debarked trees. Furthermore, the incidence of black canker caused by *B. mediterranea* had become alarming in the same years, suggesting a synergistic action between the wood-boring beetle and *B. mediterranea*. This hypothesis is supported by the high inoculum density on the colonized parts of the host trees and the probability that spores could be transported and inoculated by insects. The results of this test unequivocally demonstrated that the fungal propagules were transported specifically by *P. cylindrus* [92].

Biscopyran (**36**) belongs to the pyranopyran family, little-known as natural products but well-known as synthetic intermediates [93,94,95,96,97]. Natural pyranopyrans include brevitoxins [96] and dactomelins [97], and diplopyrone, previously reported as the main phytotoxin produced by *D. mutila*, responsible for cork oak cancer. Phenylacetic acid (**37**) was already reported as a phytotoxin produced by *Rhizoctonias* spp. [44] as had other low-molecular-weight acids, namely nitropropionic [98], oxalic [99], fumaric [100], and 3-methylthiopropionic acids [98]. The isolation of 5-methylmellein (**38**) was also not surprising, as it is the most common dihydroisocoumarin isolated from ascomycetes belonging to several genera (see *Entonaema*, *Hypoxylon*, *Xylaria*, *Resillinia*, *Poronia*, *Podosodaria*, *Hycocopra*, *Daldinia*, *Nummularia*, *Kretzschmaria*, *Camillea*, and *Penzigia*) of the Xylariaceae family. The member of this family produces structurally different toxic metabolites such as cytochalasins, naphthalene derivatives, butyrolactones, succinic acid derivatives, chromanones, and diketopiperazines. To develop a better understanding of the natural relationships within the same fungal family, several studies have been conducted considering the secondary metabolites distribution in combination with traditional taxonomic characteristics [101].

#### 2.2.2. *Biscogniauxia rosacearum*

*Biscogniauxia rosacearum*, another fungus collected from infected oak trees in the Zagros forests of Gilan-e Gharb, Kermanshah province, Iran, synthesized different metabolites. The latter were identified as (3*R*)-5-methylmellein (**38**) and nectriapyrone, (3*R*)-5-methyl-6-methoxymellein, and tyrosol (**39**–**41**, Figure 3, Table 2). Instead, *meso*-2,3-butanediol (**42**, Figure 3, Table 2) was the only phytotoxic secondary metabolite produced by a strain of the same fungus isolated for the first time as a causal agent of grapevine trunk diseases in Paveh vineyards (western Iran). Metabolites **39**–**42** were characterized as (*E*)-6-(but-2-en-2-yl)-4-methoxy-2*H*-pyran-2-one, (*R*)-8-hydroxy-6-methoxy-3,5-dimethylisochroman-1-one, 4-(2-hydroxyethyl)phenol, and butane-2,3-diol, respectively. Compounds **38**–**42** were tested for their phytotoxicity at a concentration of 5 × 10^−3^ M and 10^−3^ on leaves of *Quercus ilex* L., *Hedera helix* L., and grapevine (*Vitis vinifera* L.). The most phytotoxic compounds were *meso-*2,3-butanediol (**42**) and (3*R*)-5-methyl-6-methoxymellein (**38**). Nectriapyrone (**39**) and tyrosol (**41**) induced severe necrosis at the highest concentration using the same assays, while the compounds **38**–**42** were not toxic to *Q. helix* L. [60]. (3*R*)-5-methylmeliein (**38**) had been previously isolated as a phytotoxin from *Valsa ceratosperma*, responsible for apple canker, one of the severe diseases of this crop in the Northern regions of Japan. Four other isocoumarins were isolated from this latter pathogen and identified as (–)-5-carboxymeliein, (–)-5-hydroxymethylmeliein and (3*R*,4*S*)-*trans*- and (3*R*,4*R*)-*cis*-4-hydroxy-5-methylmeliein. All five compounds showed phytotoxic activity on detached apple shoots and lettuce seedlings [102]. Nectriaprine (**39**) was also previously produced as a phytotoxin by *Diaporthe angelicae* (anamorph *Phomopsis foeniculi*), which is a pathogen of fennel (*Foeniculum vulgare*) in Bulgaria [34]. Furthermore, compound **39** was also isolated from *Pestalotiopsis guepinii*, the causal agent of hazelnut twig blight [61], and more recently from *Diaporthe eres*, which has been implicated in GTD (Grapevine Trunk Disease) symptoms [62]. Like compound **38**, (3*R*)-5-methyl-6-methoxy-mellein (**40**) belongs to the group of 4-dihydroisocoumarins frequently found as natural products. They represent secondary metabolites involved in many biological activities, including phytotoxicity [103]. Tyrosol (**41**) is a phytotoxin produced by both plants [104] and fungi, including the grapevine pathogenic fungi *Lasiodiplodia euphorbicola*, *Lasiodiplodia hormozganensis* [63], *Neofusicoccum australe* [63], and *Neofusicoccum parvum* [63]. Tyrosol was also produced by *Diplodia seriata* (syn. *Botryosphaeria obtusa*), a pathogen of apple fruit and frog leaf [64], and it is toxic to tomato and is a quorum-sensing compound in *Candida albicans* [105]. 2,3-Butanediol is a well-known fungal and bacterial metabolite [106,107,108] and has great potential for industrial use as it is used for important biotechnological applications [109,110]. Mass production of compound 2,3-butanediol by fermentation will be important for these industrial developments [111]. Considering the stereochemical properties, 2,3-butanediol has three stereoisomers: the dextro-[L−(+)−] and levo-[D−(−)−] forms, optically active, and the optically inactive *meso*-form, such as **42**. The stereochemistry of the resulting compound depends on the producing microorganism; however, the *meso*-form is the most common stereoisomer [106,107].

#### 2.2.3. *Tubakia dryina*

**Figure 4:**
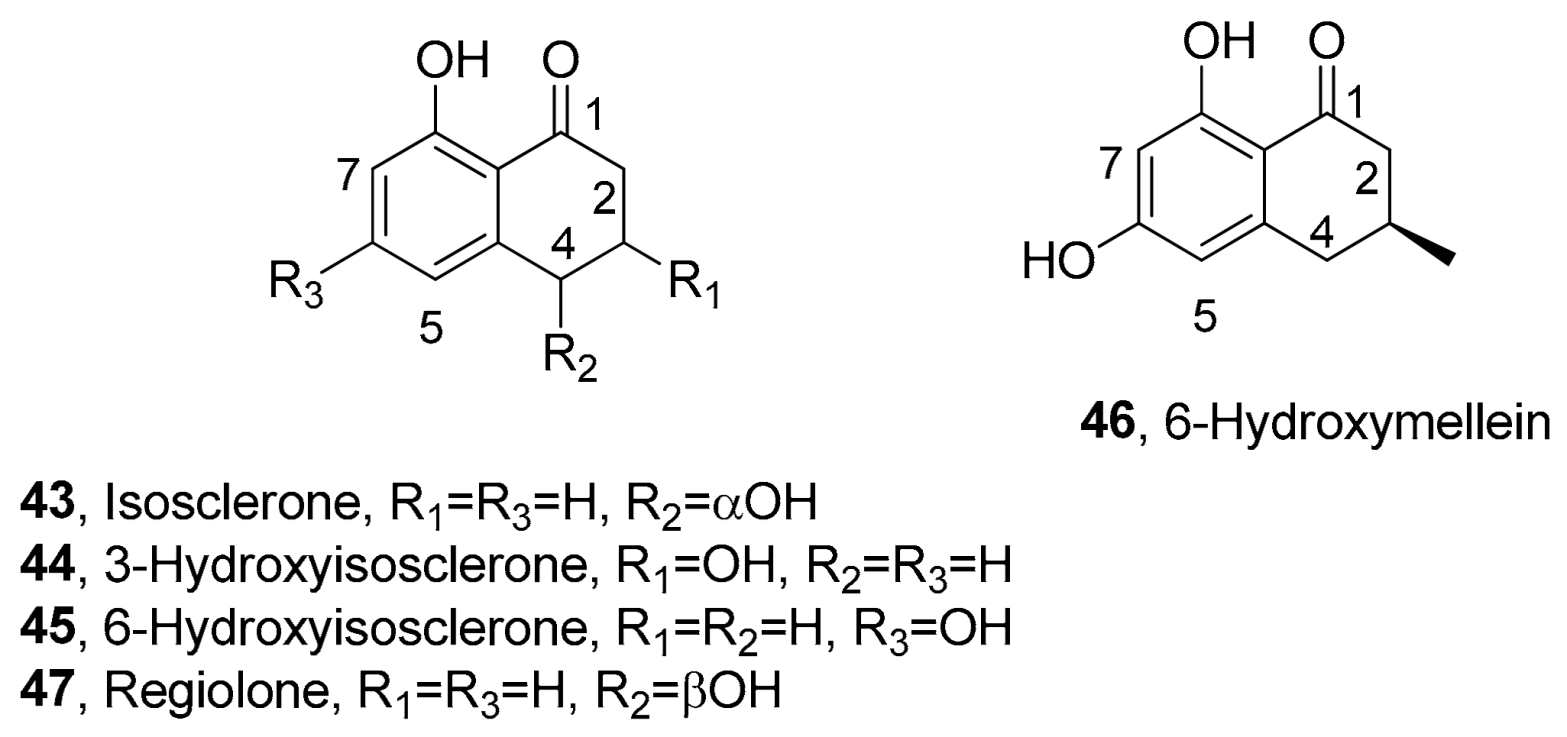
Phytotoxins produced by *Tubakia dryina.*

The fungus *Tubakia dryina* was first collected and identified in the USA, east of the Mississippi River [112]. The pathogen, which is widespread worldwide, infected several hosts [113], such as swamp oak (*Quercus palustris*) and northern red oak (*Quercus rubra* L.). *T. dryina* on red oak leaves caused roughly circular dark brown to reddish spots sized 2–5 mm, which frequently merged to form irregular patches and affected large leaf veins. The fungal culture filtrates caused moderate-sized lesions on sorghum and watercress. Some metabolites were synthesized in vitro by *T. dryina* and were isolated and identified as isosclerone, 3-hydroxyisosclerone, 6-hydroxyisosclerone, and 6-hydroxymellein (**43**–**46**, Figure 4, Table 2) [67]. The metabolites **43**–**46** were characterized as (*S*)-4,8-dihydroxy-3,4-dihydronaphthalen-1(2*H*)-one, 3,8-dihydroxy-3,4-dihydronaphthalen-1(2*H*)-one, 6,8-dihydroxy-3,4-dihydronaphthalen-1(2*H*)-one and (S)-6,8-dihydroxy-3-methyl-3,4-dihydronaphthalen-1(2H)-one, respectively. Metabolites **43**–**46** were tested using the leaf prick test on red oak leaves, which caused serious lesions along the veins that appeared within 24 h of treatment. These symptoms were very similar to those caused by *T. dryina* infection. Necrotic lesions were similarly induced on white oak and eight different weed species, including morning silica, Sida spinosa, morning glory, jimson weed, sorghum, and cress. Among these plants, morning glory and morning silica appeared to be the least sensitive. The results obtained also showed the lack of specificity of the toxins [67]. Isosclerone (**43**) was also produced by other fungi such as *Scytalidium* sp., *Sclerotinia sclerotiorum* [68], *Grignardia laricina* [69], *Botrytis cinerea* [70], *Penicillium diversum* [71], and *Discula* sp. [72]. Furthermore, compound **43** was also involved in the biosynthesis of juglone in walnut (*Juglans regia*) [114]. More recently, isosclerone together with scytalone were isolated from *Phaeoacremonium aleophilum* [63] and *Phaeomoniella chlamydospora* [73] as phytotoxins involved in esca, which is one of the most serious grapevine trunk diseases. Subsequently, studies conducted on the chocolate spot of broad bean (*Vicia faba* L.), caused by *Botrytis fabae*, highlighted an important role of regiolone (**47**, Figure 4, Table 2) [74], which is characterized as (*R*)-4,8-dihydroxy-3,4-dihydronaphthalen-1(2*H*)-one and appeared to be the C-4 enantiomer of isosclerone (**43**). The latter had also been isolated, as reported above, from *B. cinerea*, a fungus of the family Sclerotiniaceae, which is a parasite that attacks many plant species, although among the various hosts the most economically important is the grapevine, followed by all other fruits [115]. The enantiomeric relationship between compounds **43** and **47** was unambiguously resolved by ab initio computational prognostication of their theoretical optical rotatory powers and electronic circular dichroism spectra. The phytotoxic activity of the two compounds was compared by tests on broad bean (host plant of both pathogens and grapevine (host plant of *B. cinerea* only).

Regiolone and isosclerone (**47** and **43**) both showed phytotoxicity on faba bean leaves at all concentrations tested, but with regiolone (**47**) always more toxic than isosclerone (**43**). In contrast, in the bioassay on grapevine leaves, the two compounds were nontoxic at all concentrations used. These results support the hypothesis that *B. cinerea* does not utilize isosclerone when infecting the plant, but uses other phytotoxins. Instead, *B. fabae* would resort to isosclerone when attacking faba bean. *B. fabae*, which is a more aggressive faba bean pathogen than *B. cinerea*, would have further specialization to obtain a more phytotoxic metabolite such as the regiolone by modifying the C-4 configuration of isosclerone. Therefore, the C-4 configuration should be a key structural feature to impart bioactivity, resulting in (*R*) for (–)-regiolone (**47**) and (*S*) for (+)-isosclerone (**43**) [74]. These results further confirm the relationship between the absolute configuration of naturally occurring compounds and their biological activity [116]. Furthermore, 3,4,8-dihydroxytetralone (3-hydroxyisosclerone) is also produced by *T. dryina* had been reported as a metabolite of several scytalone-producing fungi [117,118], while 6-hydroxymellein has been synthesized from several other fungi [72].

#### 2.2.4. *Discula quercina*

**Figure 5:**
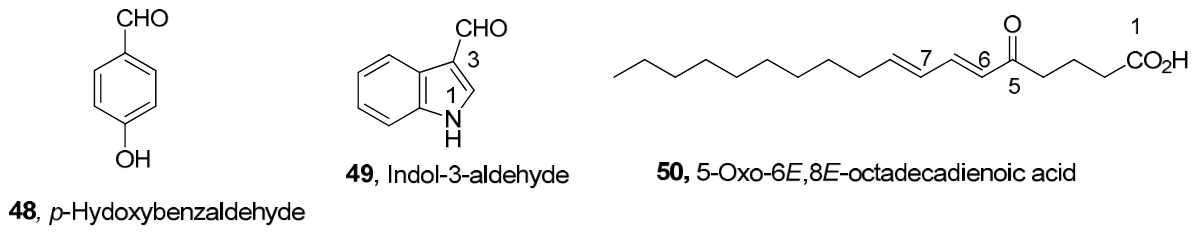
Phytotoxins produced by *Discula quercina.*

*Discula quercina* is a pathogen causing oak anthracnose disease [118]. It causes dark-brown spots on the host plant leaves surrounded by purplish margins, which, in late autumn, become large necrosis. The disease can cause cankers and dieback of the twigs, and in winter, often causes premature leaf fall. *D. quercina* sometimes seems like an endophyte without showing its presence inside the host tissues [75,119,120]. *D. quercina* synthesizes phytotoxic metabolites such as 4-hydroxybenzaldehyde, indole-3-aldehyde (1*H*-indole-3-carbaldehyde) and 5-oxo-6*E*,8*E*-octadecadienoic acid (**48**–**50**, Figure 5, Table 2). The latter, the main metabolite, is an unusual acyclic ketoacid. Indole-3-aldehyde was more toxic than 4-hydroxybenzaldehyde in a leaf puncture assay using *Q. suber* and *Q. ilex* leaves, while compound **50** was not toxic even at the highest concentration used (1 mg/mL). The phytotoxic activity of indole-3-aldehyde (**49**) was reported for the first time, although this indole derivative is a well-known microbial metabolite [29].

4-Hydroxybenzaldehyde (**48**) is a known fungal phytotoxin produced by pathogens of important agricultural crops (e.g., apple, leek, onion, and grapevine) [29,72,73,121]. It was also produced as a phytotoxin by *Ceratocystis* spp., causing pine blue spot [76], by a phytopathogenic species of *Monilia* sp. [77], by *Pythium aphanidermatum*, responsible for *Pythium* red blight, and *Pythium* on bentgrass [78]. Recently, 4-hydroxybenzaldehyde (**48**) was isolated from *Phoma exigua* var. *exigua* and proposed as a mycoherbicide for the biocontrol of the perennial weeds *Sonchus arvensis* L. and *Cirsium arvense*, together with cytochalasins B, F, Z2, and Z3 and the deoxaphomin (L.) [79]. Cytochalasins exhibited moderate phytotoxicity when assayed on host plant leaves, while 4-hydroxybenzaldehyde (**48**) showed no toxicity. This result is consistent with that observed for bentgrass [63], but contrasted with the toxicity observed on the leaves of 17 apple cultivars and eight weed species [57]. These different effects could be due to the different sensitivity of the plants and/or the different concentrations used, as reported for other fungal phytotoxins [63,79].

Indole-3-aldehyde (**49**) represents an indole-3-acetic acid (IAA) degraded derivative; it is a product of an alternative degradation of IAA with respect to its oxidation induced by horseradish peroxidase [122]. Compound **49** is frequently reported as a bacterial and fungal metabolite [123,124]. It with IAA, the corresponding two different IAA-lysine conjugates, and cytokinins, was produced by *Pseudomonas savastanoi* pv. *savastanoi*, the pathogen involved in olive canker [124]. *Pantoae agglomerans*, a common epiphyte of many plants, had been frequently found together with *P. syringae* pv. *savastanoi* in apparently intact young olive nodes. *P. agglomerans* synthesized indole-3-aldehyde along with indole-3-ethanol and IAA [124]. Although the involvement of IAA in pathogenesis has been demonstrated for many phytopathogenic bacteria, the role of indole-3-aldehyde as a phytotoxin remains to be fully elucidated [125]. Furthermore, most of these indole and cytokinin derivatives have shown other different biological properties, including antimicrobial and anticancer activity [126,127].

5-Oxo-6*E*,8*E*-octadecadienoic acid (**50**) was first isolated as a fungal metabolite from *D. quercivora*, even if the production of a fatty acid from phytopathogenic fungi is common. Previously, compound **50** was obtained from the extracts of potato tubers collected in the Peruvian Andes (*Lepidium meyenii* Walpers) [128,129].

#### 2.2.5. *Alternaria tenuissima*

**Figure 6:**
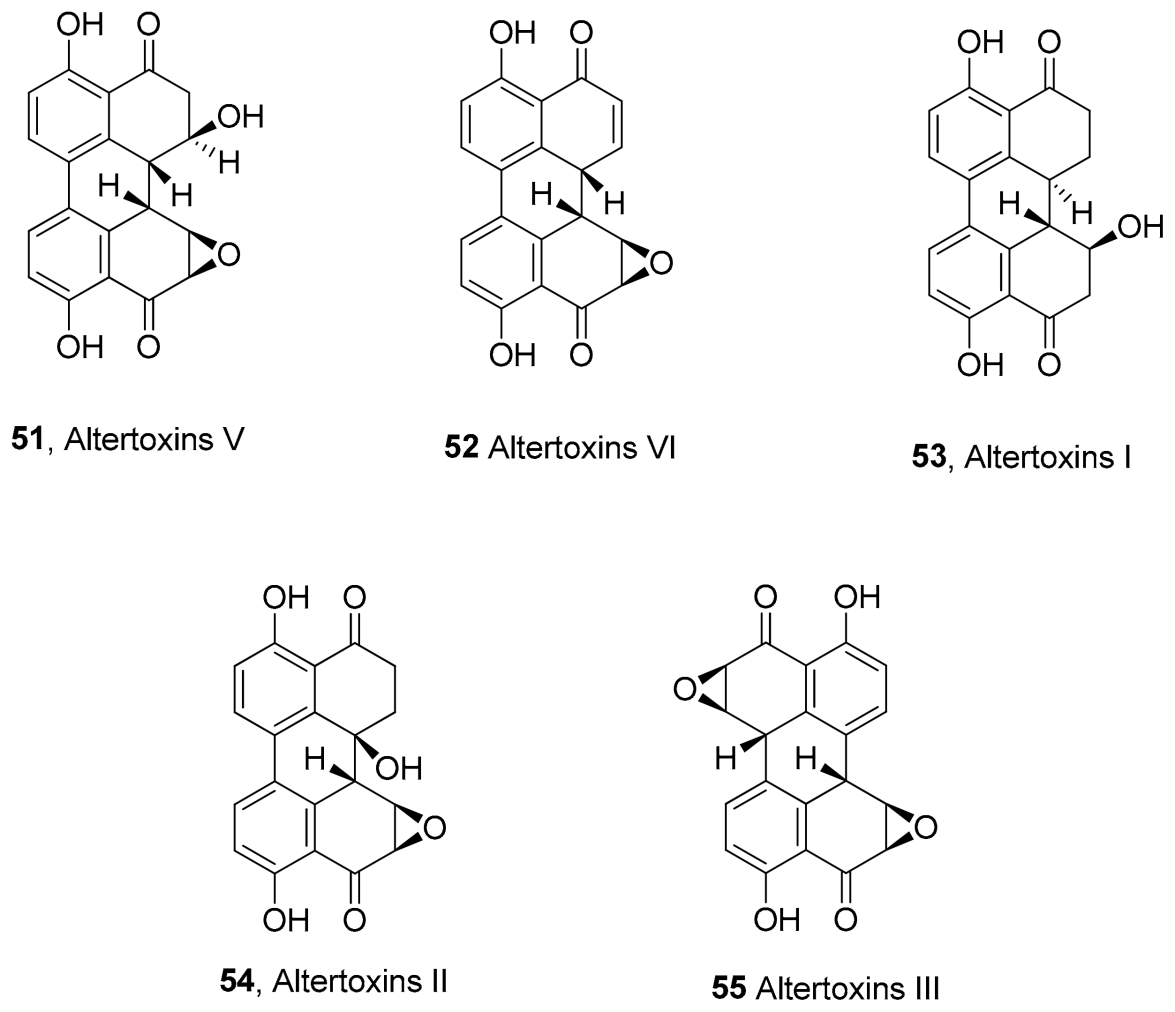
Phytotoxins produced by *Alternaria tenuissima.*

Altertoxins V and VI (**51** and **52**, Figure 6, Table 2) were isolated from *Alternaria tenuissima* inhabiting oak (*Quercus emoryi*) stem tissue, along with the known altertoxins I, II, and III (**53**–**55**, Figure 6, Table 2) [80]. Compound **51** and **52** were characterized as (7a*R*,8a*R*,8b*S*,8c*R*,9*R*)-1,6,9-trihydroxy-8b,8c,9,10-tetrahydroperyleno [1,2-b]oxirene-7,11(7a*H*,8a*H*)-dione and (7a*R*,8a*R*,8b*S*,8c*R*)-1,6-dihydroxy-8b,8c-dihydroperyleno [1,2-b]oxirene-7,11(7a*H*,8a*H*)-dione, respectively. The three analogs **53**–**55** were similarly characterized as (1*S*,12a*S*,12b*S*)-1,4,9-trihydroxy-1,12,12a,12b-tetrahydro perylene-3,10(*2H,*11*H*)-dione, (7a*R*,8a*R*,8b*S*,8c*S*)-1,6,8c-trihydroxy-8b,8c,9,10-tetrahydro peryleno [1,2-b]oxirene-7,11(7a*H*,8a*H*)-dione, and (1a*R*,1b*S*,5a*R*,6a*R*,6b*S*,10a*R*)-4,9-dihydroxy-5a,6a,6b,10a-tetrahydroperyleno [1,2-b:7,8-b′]bis(oxirene)-5,10(1a*H*,1b*H*)-dione, respectively. *A. tenuisiima*, which is a saprophytic pathogen that infects different plants [130,131], is widespread worldwide [132,133]. Alternariol, its monomethyl ether, altenuene, tenuazonic acid, all toxic metabolites, and the caseinolytic enzyme protease and beta 1,3-glucanase have been produced by the same fungus [134,135,136,137,138]. Some perylene oxides and alterperylenols, which show antibacterial, antifungal, mutagenic, and phytotoxic activities, have been isolated from several *Alternaria* species [139,140,141,142,143,144]. Previously, altertoxin II [126,145] and altertoxin IV [146] had been produced by *A. tenuissima* isolated from the medicinal plant *Tribulus terrestris* [147]. All compounds were tested as inhibitors of HIV-1 viral replication in infected A3.01 cells. Compounds **50** and **52**–**54**, assayed at 1.5 μg/mL, almost completely inhibited (97–99%) viral replication more than 20 μM AZT (azidothymidine), used as a positive control, while compound **51** caused only 33% inhibition. The reduced activity of the latter was probably caused by its instability in the cell culture medium. Furthermore, altertoxin V (**50**) showed an IC_50_ (50% Inhibitory Concentration) of 0.09 μM, and almost complete inhibition of viral replication at 0.50 μM. Altertoxins I, II, and III (**52**–**54**) exhibited higher IC_50_ values of 1.42, 0.21, and 0.29 μM, respectively, with nearly complete inhibition of viral replication occurring at 2.20, 0.30, and 1.50 μM, respectively. Considering the structure–activity relationships, these data suggest that epoxytetralone and [1,10-biphenyl]-4,40-diol were important structural features for the antiviral activity [80].

#### 2.2.6. *Raffaelea quercivora*

**Figure 7:**
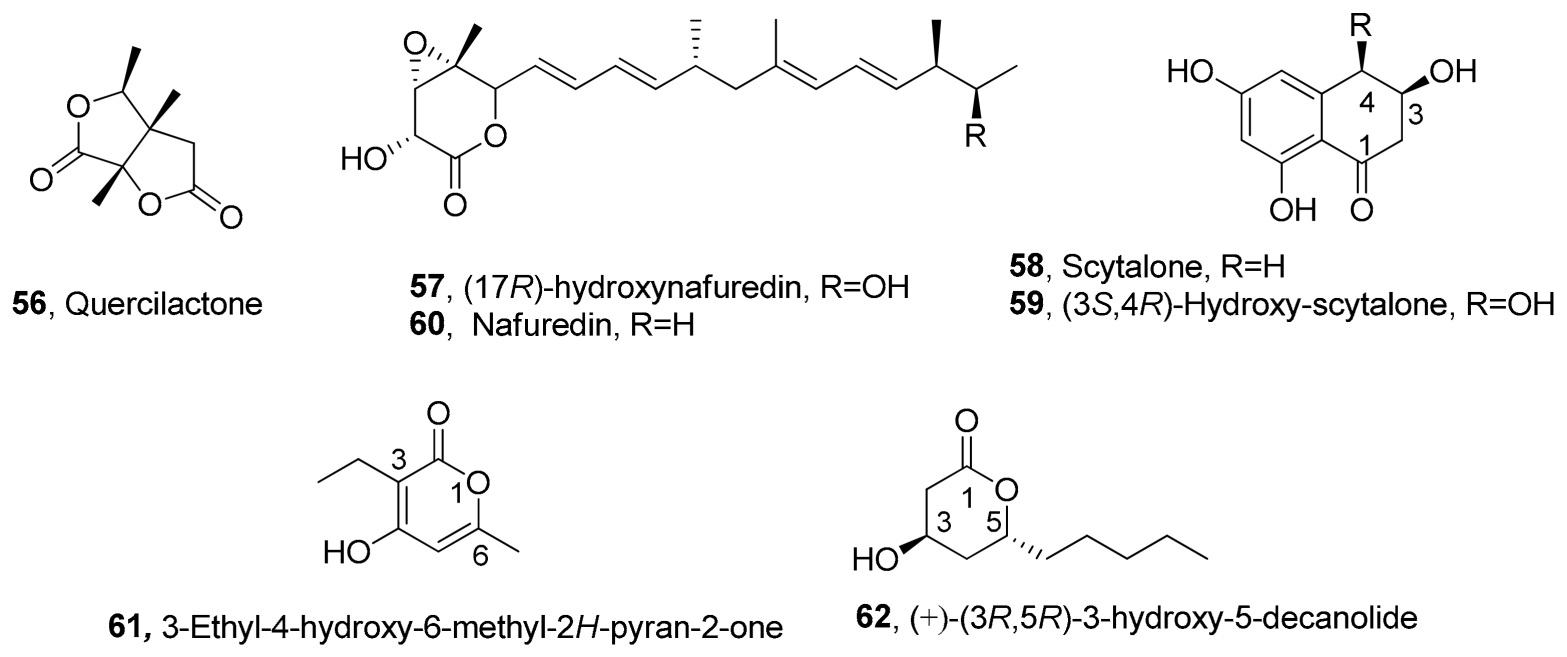
Phytotoxins produced by *Raffaelea quercivora.*

Quercilactone A and (17*R*)-hydroxynafuredin (**56** and **57**, Figure 7, Table 2), a new bis-γ-lactone, as well as a new α-pyrone, were isolated from *Raffaelea quercivora* and haracterized as ((3a*S*,4*S*,6a*S*)-3a,4,6a-trimethyldihydrofuro [3,4-b]furan-2,6(3*H*,6a*H*)-dione;and (1*R*,5*R*,6*S*)-5-hydroxy-2-((1*E*,3*E*,5*R*,7*E*,9*E*,11*R*,12*R*)-12-hydroxy-5,7,11-trimethyltrideca-1,3,7,9-tetraen-1-yl)-1-methyl-3,7-dioxabicyclo [4.1.0]heptan-4-one, respectivelyAlso isolated from the same fungus were scytalone ((*R*)-3,6,8-trihydroxy-3,4-dihydronaphthalen-1(2*H*)-one), (3*S*,4*R*)-hydoxy-scytalone ((3*S*,4*R*)-3,4,6,8-tetrahydroxy-3,4-dihydronaphthalen-1(2*H*)-one), nafuredin ((1*R*,5*R*,6*S*)-5-hydroxy-1-methyl-2-((1*E*,3*E*,5*R*,7*E*,9*E*,11*S*)-5,7,11-trimethyltrideca-1,3,7,9-tetraen-1-yl)-3,7-dioxabicyclo [4.1.0]heptan-4-one), 3-ethyl-4-hydroxy-6-methyl-2*H*-pyran-2-one, and (+)-(3*R*,5*R*)-3-hydroxy-5-decanolide (**58**–**62**, Figure 7, Table 2) [81]. The ambrosia beetle *Platypus quercivorus* is the vector of *R. quercivora,* which caused the oak wilt (JOW, *Quercus crispula*) disease in Japan [148,149]. JOW was first recognized in Japan more than 150 years ago and is a severe problem in abandoned coppice forests. This disease, since the 1980s, has spread to several areas [150] and, up to 2009, was diffused along the coasts of Japan’s Sea and particularly in its northern part [151]. The phytotoxicity of compounds **56**, **58** and **62** was assayed at concentrations ranging from 10 to 100 μg/mL on the lettuce seedling (*Lactuca sativa* L.) seed germination and root and shoot length. With respect to the negative control tested at the same concentration, compounds **56**, **58**, and **62** inhibited the growth of roots to 50.4%, 54.8%, and 53.0%, respectively [81]. Scytalone and the 3*S*,4*R*-hydoxy-scytalone derivative (**58** and **59**) are well known as fungal metabolites that are produced from several species, such as *Leptographium qinlingensis* [82], *Pyricularia oryzae* [83], *Phaeosphaeriae* sp. BCC8292 [84], and *Ceratocystis fimbriate* [85]. Scytalone and the other related polyketide naphthalenones, such as isosclerone (**58** and **43**), were also intermediates in the biosynthesis of fungal melanin [152] and showed, as reported above, significant phytotoxicity on grapevine leaves as produced as phytotoxins of the pathogen *P. aleophilum* and *P. chlamydospora,* which together with *Fomitiporia mediterranea*, a wood-degrading fungus involved in esca disease, are responsible for the serious esca grapevine disease [63]. However, none of the compounds isolated from *R. quercivora* showed phytotoxicity toward *Quercus crispula* [81].

#### 2.2.7. Fungal Pathogen for Iranian Oaks

Oaks are the most widespread trees in the Zagros forests (Iran) and represent a significant economic and environmental heritage. The decline of oaks in this region of Iran, due to biotic and abiotic stresses, including climate change, drought, fires, air pollution, irresponsible logging and mismanagement, has been spreading rapidly over the past two decades, causing serious ecological and economic damages. Pathogenic fungi, among biotic agents, attack stressed trees and cause cankers, gummosis, necrosis of vascular tissue, desiccation, and partial and total wilting. Several fungal species have been associated with the decline of woody plants, including coelomycetes fungi, belonging to the class *Dothideomycetes*. Among this class, the most widespread species belong to the group of *Botryosphaeriaceae* (e.g., *Botryosphaeria*, *Diplodia*, *Lasiodiplodia*, and *Neofusicoccum* spp.) *Cytosporaceae* (e.g., *Cytospora* spp.), *Gnomoniaceae* (e.g., *Discula* and *Gnomoniopsis* spp.), and *Graphostromataceae* (e.g., *Biscogniauxia* spp.). In particular, the fungi inducing oak dieback in the central part of this forest belong to 24 species, which also include eight coelomycete species in the genera *Cytosporaceae*, *Diatrypaceae*, *Biscogniauxia*, and *Phoma-like* [153].

**Figure 8:**
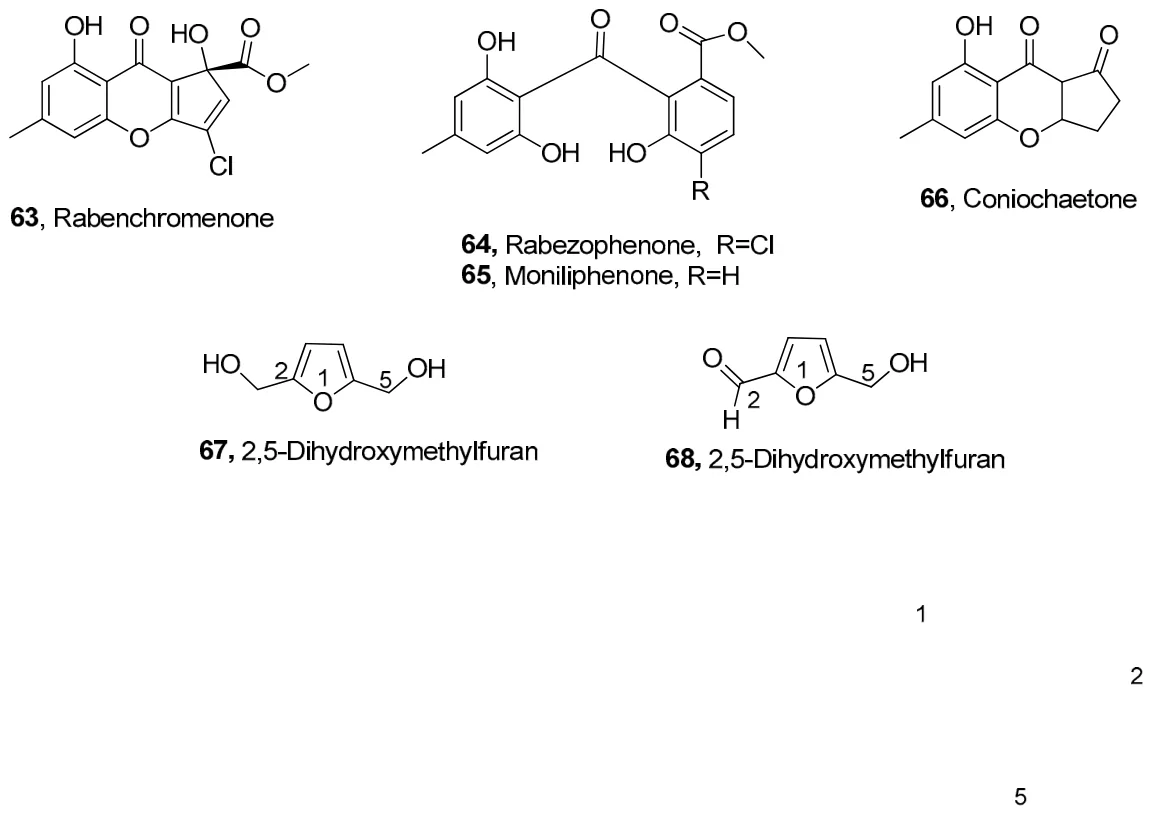
Phytotoxins produced by *Fimetariella rabenhorstii* and *Stilbocrea macrostoma*, virulent fungi collected in Zagros forest, Iran.

Some fungi were associated with oak (*Quercus brantii*) disease causing decline and wood necrosis [86]. These pathogens were grown in vitro to test their capacity to synthesize phytotoxins. Among them, *Fimetariella rabenhorstii* was recognized. This pathogen was also found as an endophytic fungus coupled with *Aquilaria sinensis* in mainland China [154]. From the organic extract of *F. rabenhorstti* culture filtrates, phytotoxic tetrasubstitutedchromen-4-one and a hexasubstituted benzophenone, named rabenchromenone and rabenzophenone A, (**63** and **64**, Figure 8, Table 2), respectively, were isolated along the related moniliphenone and coniochaetone A (**65** and **66**, Figure 8, Table 2), Compounds **63** and **64** were characterized as methyl 3-chloro-1,8-dihydroxy-6-methyl-9-oxo-1,9-dihydrocyclopenta[*b*]chromene-1-carboxylate and methyl-l4-chloro-2-(2,6-dihydroxy-4-methylbenzoyl)-3-hydroxybenzoate, respectively [86], while compounds **65** and **66** were previously identified as methyl 2-(2,6-dihydroxy-4-methylbenzoyl)-3-hydroxybenzoate and 8-hydroxy-6-methyl-3,3a-dihydrocyclopenta[b]chromene-1,9(2*H*,9a*H*)-dione, respectively. The phytotoxicity of all metabolites (**62**–**67**) was evaluated by a leaf puncture bioassay on holm oak and tomato leaves at 1 mg/mL and was found to be phytotoxic, causing on both plants a necrosis diameter ranging between 0.2 and 0.5 cm. In particular, rabenzophenone A (**64**) exhibited the highest phytotoxicity on both plants, causing significant necrostic lesions (0.7 and 0.5 cm on holm oak and tomato leaves, respectively). Compounds **65** and **66** were isolated for the first time from *F. robenhorstii* as phytotoxic metabolites [86]. While cyclopentabenzopyran-4-ones are rare as natural compounds, 4*H*-benzochroman-4-ones are widely distributed as fungal metabolites [29]. In addition, coniochaetones A-I were previously reported as fungal metabolites [155,156,157]. Benzophenones are included in a group constituted by more than 300 natural substances with differently functionalized carbon skeletons. These latter have been isolated from plants and fungi and exhibit several biological activities, including antifungal, antimicrobial, anti-HIV, antiviral, antioxidant, and cytotoxic [158].

*Stilbocrea macrostoma* is another virulent fungus isolated from branches of oak trees (*Q. brantii*) in Zagros forest, in Paveh, Kermanshah Province, Iran, and inducing wood necrosis and decline symptoms on the host plant. This pathogen was also recognized on 12 different woody plants in New Zealand (https://nt.ars-grin.gov/fungaldatabases; URL accessed on 20 August 2019) and on another host plant in Sri Lanka [159]. *S. macrostoma* produced two furan phytotoxins together with tyrosol (**41**), already described above [65]. The two furan derivatives were characterized as 5-ydroxymethylfuraldehyde and 2,5-dihydroxymethylfuran (**67** and **68**, Figure 8, Table 2) [65]. Compounds **41**, **67** and **68** were tested for their phytotoxicity by leaf puncture assay on holm oak (*Q. ilex* L.) and tomato (*Lycopersicon esculentum* L.) leaves, at a concentration of 5.0, 1.0 and 0.1 mM. All metabolites exhibited stronger phytotoxicity on oak at all concentrations used, while on tomato leaves they had no toxicity. In particular, compounds **67** and **68,** at two concentrations (5.0 and 1.0 mM), caused significant necrosis on Holm oak leaves. However, at the lower concentration, the phytotoxic effects for both compounds appeared reduced, and that of **41** was always lower compared to previous ones [65].

2,5-Dihydroxymethylfuran (**67**), first isolated from *Xylaria longipes*, also showed antifungal activity against *Nematospora coryli* [160]. Subsequently, compound **67** was isolated from *Colletotrichum acutatum*, a phytopathogenic fungus that induces anthracnose on a wide range of plants [87], from *Lacrymaria velutina*, a fungus that inhibits nitric oxide synthase [88], and from *Paecilimyces* sp., a marine filamentous fungus, with antimicrobial activity [89].

5-Hydroxymethyl-2-furaldehyde (**68**) was previously obtained from cultures of *Tylophilus felleus*, which stimulated *Holarrhena antidysenterica* callus growth [90]. Compound **68** was also synthesized by *Pleurotus ferulae*, an edible mushroom, and exhibited nematicidal activity against *Bursaphelenchus xylophilus* and *Pangrellus redivivus* [91]. Furthermore, when compound **68** was isolated from the marine fungus *Penicillium chrysogenum* HGQ6, it showed cytotoxic activity against BGC823 cells [161]. Recently, it was also found among the metabolites synthesized by *Bacillus subtilis*, and had anti-biofilm activity against *Candida albicans*, with a concentration-dependent effect [162].

Tyrosol (**41**), previously described as a phytotoxin [63,64], was recently isolated from filter cultures *of Colletotrichum lupini*, the causal agent of lupin anthracnose (*Lupinus albus* L.), which is a destructive seed-borne disease appearing on stems and pods of the host plant [66].

## 3. Conclusions

This manuscript reports on fungal diseases of numerous oak trees and the serious damage they cause to the forest heritage, which is important for the survival of all living species on Earth. The severe damages on wood industry and nurseries was also evaluated. Pathogen fungi play an important role in inducing oak decline, which is one of the most relevant forest diseases globally; the pathogens also affect disease development and its global distribution [9]. The phytotoxins, which are fungal secondary metabolites, play a significant role in plant disease induction, and those produced by pathogens of different oak species in various world regions are described in depth. In particular, their chemical and biological properties were extensively reported. For some phytotoxins, the enatioselective synthesis and the structure–biological activity relationships (SAR) are also described. In addition, for the *Diplodia* species involved in oak diseases, the comparison between their secondary metabolite profile and taxonomic characteristics with those of other close pathogens of forest plants was discussed. Phytotoxins produced are involved in severe diseases of agricultural, forest, and weed plants that cause heavy economic losses and serious environmental impact. causing heavy damage to agricultural production and the agri-food industry and environmental heritage. Their isolation and chemical and biological characterization, as well as the results of SAR and the modes of action studies, can help to investigate the interaction between plant and pathogen and the complex pathogenic process in depth. The SAR study performed on several phytotoxins with different carbon skeletons and functionalities, including those reported in the present review, and their natural analogs and some key semisynthetic derivatives, has shown that the presence of an α,β-unsaturated carbonyl or an epoxy group, which allowed the attachment of a nucleophilic group by Michael addition or nuclephilic substitution, respectively, are frequently perceived as an important structural feature to impart biological activity. Consequently, the results of these studies can allow for evaluating the potential practical use of phytotoxins in other fields. Phytotoxins could have some potential applications: (1) to develop rapid, simple, and specific methods for plant disease recognition; (2) to select plant varieties resistant to the disease; (3) to find new natural compounds, particularly those with new carbon skeletons and/or new modes of action that would overcome the resistance developed by pathogens; (4) to develop biopesticides based on natural substances that, included in suitable formulations, could be applied in agriculture [9] and to design new drugs inspired for their application in medicine [57,163,164].

## Figures and Tables

**Table 1:** Phytotoxins produced by *Diplodia* spp. pathogens of oak (*Quercus*).

Phytotoxin	Chemical Family	Fungus	Host Plant	Other Biological Activity	Ref.
Diplopyrone (**1**)	Non aromatics Heterocyclics	*Diplodia mutila* *Diplodia coticola*	Oak (*Quercus ruber*)		[26,29]
Diplobifuranylone A (**5**)	Furans	*corticola*	“		[29]
Diplobifuranylone B (**6**)	“	“	“		“
(3*S*,4*R*)-*trans*-4-Hydroxymellein (**7**)	Naphthalenones	*D. corticola* *Diplodia africana* *Diplodia subglobulosa*	Oak Phoenician juniper (*Juniperus phoenicea* L.) Ash (*Fraxinus excelsior* L.)		[29]
(3*R*,4*R*)-*cis*-4-Hydroxymellein (**8**)	“	“	“		“
Sapinofuranone B (**9**)	Furans	*D. corticola* and *Sphaeropsis sapinea* *Diplodia sapinea*	Oak cypress (*Cupressus macrocarpa)* *Conifers*		[29]
(*S*,*S*)-Enantiomer of **9** (**10**)	“	*D. corticola Acremonium strictum*	“		[35]
Sphaeropsidin A (**11**)	Diterpenes	*D. corticola, Diplodia cupressi* *Diplodia quercivora* *D. africana* *D. subglobulosa*	Oak Cypress (*Cupresus sempervirens* L.) Phoenician juniper Ash	Antimicrobial, insecticidal, herbicidal anticancer activity	[29]
Sphaeropsidin B (**12**)	“	*D. corticola*	Oak Cypress		[29]
Sphaeropsidin C (**13**)	“	*D. corticola* *D. quercivora* *D. subglobulosa*	Oak Ash		[29]
Sapinofuranone A (**14**)	Furans	*Sphaeropsis sapinea* *D. sapinea*	Oak Cypress (*Cupressus macrocarpa*) Conifers		[29]
Diplofuranone A (**15**)	“	*D. corticola*	Oak		[29]
Diplofuranone B (**16**)	“	“	“		“
Sphaeropsidin G (**17**)	*nor*-Diterpenes	*D. corticola D. olivarum*	Oak Olive (*Olea europea*) Lentisk (*Pistacia lentiscus*)	Zootoxic	[29,36]
Diplobifuranylone D (**18**)	Furans	*D. corticola*	Oak	“	[29]
4-Hydroxyscytalone (**19**)	Naphthalenones	“	“	“	“
Diorcinol (**20**)	Phenols	“	“	Antifungal	“
Diplopimarane (**21**)	*nor*-Diterpenes	*Diplodia quercivora*	Oak (*Quercus canariensis*)	Antifungal Zootoxic	[29]
(+)-*epi*-Epoformin (**22**)	Cyclohexene oxides	“	“	“	[29,37]
Afritoxinone A (**23**)	Furan-pyrones	*Diplodia africana*	*P. juniper*		[29]
Afritoxinone B (**24**)	“	“	“		“
Oxysporone (**25**)	“	“	“		“
(−)-(2*R*,3*R*,4*R*)-*epi*-spheropsidone (**26**)	Cyclohexene oxides	*D. africana* *D. subglobulosa*	*P. juniper* Ash		[29,38]
*R*-(−)-Mellein (**27**)	Naphthalenones	*D. africana* *D. subglobulosa* *D. sapinea* *S. sapinea*	*P. junipe* *Ash* Conifer, Pine (*Pinus radiata*)		[29]
Diplodialide A (**28**)	Nonenolides	*D. sapinea*	Conifers		[29]
Diplodialide B (**29**)	“	“		“	“
Diplodialide C (**30**)	“	“		“	“
Diplodialide D (**31**)	“	“		“	“
Sapinopyridione (**32**)	Aromatic heterocycle	“	“		“
Pinofuranoxin A (**33**)	Furans	“	Pine (*Pinus pinaster*)	Antifungal Zootoxic	“
Pinofuranoxin B (**34**)	“	“	“		“
Diplofuranoxin (**35**)	“	*Diplodia subglobulosa*	Ash		“

“ means the species is the same as above.

**Table 2:** Phytotoxins produced by other pathogens of oak (*Quercus*).

Phytotoxin	Chemical Family	Fungus	Host Plant	Other Biological Activity	Ref.
Biscopyran (**36**)	Pyranopyran	*Biscogniauxia mediterranea*	Oak (*Quercus suber* L.)		[29]
Phenylacetic acid (**37**)	Aromatic acid	“	“		“
(3*R*)-5-Methylmellein (**38**)	Naphthalenones	*B. mediterranea* *Biscogniauxia rosacearum* *Valsa ceratosperma*	Oak “ Apple (*Malus domestica*)		[29,60]
Nectriapyrone (**39**)	Pyrones	*B. rosacearum Diaporthe angelicae* *Pestalotiopsis guepinii* *Diaporthe eres*	Oak fennel (*Foeniculum vulgare*) Halzenut (*Corylus avellana*) Grapevine (*Vitis vinifera*)		[34,60,61,62]
(3*R*)-5-Methyl-6-methoxymellein (**40**)	Naphthalenones	*B. rosacearum*	Oak		[60]
Tyrosol (**41**)	Phenols	*B. rosacearum* *Lasiodiplodia euphorbicola Lasiodiplodia hormozganensis* *Neofusicoccum australe Neofusicoccum parvum* *Diplodia seriata* *Stilbocrea macrostoma* *Colletotrichum lupini*	Oak Grapevine “ “ “ Apple Oak *Stilbocrea macrostoma* Lupin (*Lupinus* spp.)	Quorum-sensing in *Candida albicans*	[60,63,64,65,66]
*meso*-2,3-Butanediol (**42**)	Alcohols	*B. rosacearum*	Grapevine		[60]
Isosclerone (**43**)	Naphthalenones	*Tubakia dryina* *Scytadilium* sp. *Sclerotinia sclerotiorum*, *Grignardia laricina Botrytis cinerea Penicillium diversum and Discula* sp. *Phaeoacremonium aleophilum Phaeomoniella chlamydospora*	Oak (*Quercus palustris* and *Quercus rubra* L.) Grapevine		[63,67,68,69,70,71,72,73]
3-Hydroxyisosclerone (**44**)	“	*T. dryina*	Oak (*Quercus palustris* and *Quercus rubra* L.)		[67]
6-Hydroxyisosclerone (**45**)	“	*“*	“		“
6-Hydroxymellein (**46**)	“	*“*	“		“
Regiolone (**47**)		*Botrytis fabae*	Broad bean (*Vicia faba* L.)		[74]
4-Hydroxybenzaldehyde (**48**)	Aromatic compounds	*Discula quercina* *Ceratocystis* spp. *Monilia* sp. *Pythium aphanidermatum* *Phoma exigua* var*. exigua*	Oak (Quercus) Pine (*Pinus pinaster*) Bentgrass (*Agrostis* sp. Perennial thistles (*Sochus arvensis* L. *Cirsium arvense*)		[75,76,77,78,79]
Indole-3-aldehyde (**49**)	Indoles	*D. quercina*	Oak		[75]
5-Oxo-6*E*,8*E*-octadecadienoic acid (**50**)	Fatty Acids	“	“		“
Altertoxins V (**51**)	Perylenequinones	*Alternaria tenuissima*	Oak (*Quercus emoryi*)		[80]
Altertoxins VI (**52**)	“	“	“		“
Altertoxins I (**53**)	“	“	“		“
Altertoxins II (**54**)	“	“	“		“
Altertoxins III (**55**)	“	“	“		“
Quercilactone A (**56**)	Furan	*Raffaelea quercivora*	Oak (*Quercus crispula*)		[81]
(17*R*)-Hydroxynafuredin (**57**)	Pyrones	“	“		“
Scytalone (**58**)	Naphthalenone	*Oak* *Leptographium qinlingensis Pyricularia oryzae Phaeosphaeriae* sp. *BCC8292 Ceratocystis fimbriata*	“		[63,81,82,83,84,85]
(3*S*,4*R)*-Hydoxy-scytalone (**59**)	“	“	“		“
Nafuredin (**60**)	Pyrones	*Raffaelea quercivora*	Oak		[81]
3-Ethyl-4-hydroxy-6 methyl-2*H*-pyran-2-one (**61**)	“	“	“		“
(+)-(3*R*,5*R*)-3-Hydroxy-5-decanolide (**62**)	“	“	“		“
Rabenchromenone (**63**)	O-Heteroaromatic compounds	*Fimetariella rabenhorstii*	Oak *(Quercus brantii*)		[86]
Rabenzophenone A (**64**)	Aromatic compounds	“	“		“
Moniliphenone (**65**)	“	“	“		“
Coniochaetone A (**66**)	O-Heteroaromatic compound	“	“		“
5-Hydroxymethylfuraldehyde (**67**)	Furans	*S. macrostoma* *Xylaria longipes* *Colletotrichum acutatum* *Paecilimyces* sp.	“		[87,88,89]
2,5-Dihydroxymethylfuran (**68**)	“	*S. macrostoma* *Tylophilus felleus* *Pleurotus ferulae*	“	Nematocide, cytotoxic, antimicrobial	[65,90,91]

“ means the species is the same as above.

## Data Availability

No new data were created or analyzed in this study. Data sharing is not applicable to this article. The search strategy focused on the literature that directly addressed the main topic. Major electronic databases, search engines, SciFinder, and Google Scholar were used for articles relevant to the topic. The search involved specific combinations of keywords using terms such as: quercus, disease, pathogenic fungi, and phytotoxins. This methodology yielded a broad selection of articles on the subject, the content of which was briefly but thoroughly described using chronological order in each fungal subgroup.
